# Interventional Mental Health: A Transdisciplinary Approach to Novel Psychiatric Care Delivery

**DOI:** 10.7759/cureus.43533

**Published:** 2023-08-15

**Authors:** Jonathann Kuo, Tabitha Block, Megan Nicklay, Brandon Lau, Marcel Green

**Affiliations:** 1 Regenerative and Anti-Aging Medicine, Hudson Health, New York, USA; 2 Research, Hudson Health, New York, USA; 3 Pain Management, Hudson Health, New York, USA; 4 Psychiatry, Hudson Health, New York, USA

**Keywords:** iv ketamine infusion, autonomic dysregulation, post traumatic stress disorder (ptsd), treatment of depression, treatment-resistant depression, major depressive disorder (mdd), generalized anxiety disorder (gad), repetitive transcranial magnetic stimulation (rtms), ketamine infusion, stellate ganglion block (sgb)

## Abstract

Mental health disorders are among the most common health conditions in the United States. Traditional clinical treatments rely on psychiatric counseling and, in many cases, prescription medications. We propose an innovative model, Interventional Mental Health, which employs a combination of modalities through a multifaceted approach to treat conditions that have exhibited limited responsiveness to traditional methods and individuals afflicted with multiple comorbidities simultaneously. We hypothesize that creating a unique treatment algorithm combining current therapeutic modalities such as Stellate Ganglion Blocks (SGB), Transcranial Magnetic Stimulation (TMS) therapy, and ketamine therapy, within a consolidated timeframe, will yield synergistic outcomes among patients presenting with comorbid post-traumatic stress disorder (PTSD), depression, and/or anxiety.

## Introduction and background

Interventional Mental Health (IMH) is a novel, multidisciplinary approach to the delivery of mental health services to psychiatric populations. Psychiatry is a discipline characterized by its specialized training, emphasizing psychopharmacology and psychotherapy domains [1]. These conventional interventions require little in the way of infrastructure or technology, and the technical and organizational requirements have more in common with the “Freudian Couch'' than the modern operating room. However, with the rapid pace of complex interventional care increasing, we present an overview of the opportunities and challenges for transdisciplinary care in psychiatric conditions and co-occurring presentations.

Sleep medicine is a medical subspecialty that includes doctors with prior training in pulmonary critical care, psychiatry, and neurology. The model we present for IMH is similar. Our model is based on a novel service design bringing together psychiatry and anesthesia. An interventional approach to psychiatric disorders requires knowledge and technical skills from psychiatry, pain management, anesthesiology, and even neurosurgery to deploy innovative and logistically intricate management strategies for mental health disorders. Common factors unite the systems and training needs for new treatments for post-traumatic stress disorder (PTSD), major depressive disorder (MDD), obsessive-compulsive disorder (OCD), generalized anxiety disorder (GAD), and more. 

This review presents the integrated model for deploying three treatments initially: Stellate Ganglion Blocks (SGB) for PTSD and GAD, intravenous ketamine therapy for MDD, and Transcranial Magnetic Stimulation (TMS) for MDD and OCD. As GAD and PTSD are among the most common comorbid mental health conditions and have an estimated comorbidity rate of 50% in civilian populations and up to 90% in veteran populations, and approximately 50% of patients with PTSD also suffer from MDD, we present the evidence supporting the efficacy of these treatment modalities and discuss the rationale for combining what we consider prototypical interventions with the novel combination of several psychedelic treatments to improve clinical outcomes in myriad psychiatric disorders [2-4]. Further, our research site, which offers an Interventional Pain Fellowship, has begun the work of providing an integrated training experience for psychiatric conditions. Further, our research site, which offers an Interventional Pain Fellowship, has begun the work of providing an integrated training experience for psychiatric conditions. We propose an IMH Fellowship to establish a transdisciplinary training framework for the next generation of subspecialists.

## Review

Conditions with new treatments opportunities

Generalized Anxiety Disorder (GAD)

GAD is among the most common psychiatric conditions, with a preponderance of symptoms suggesting the strong role of the brain-body connection in its pathophysiology [5]. GAD presents with characteristic psychological symptoms such as excessive, persistent, unrealistic fear, worry, and feeling overwhelmed [5-7]. These “thought and feeling” psychiatric symptoms present with somatic symptoms: non-specific physiological symptoms are diagnostic criteria (increased heart rate, shortness of breath, chest pain, hyperventilation, sweating, nausea, trembling). Moreover, cognitive symptoms (fear of losing control, fear of physical injury, confusion) and behavioral symptoms (restlessness, acing) are pathological in the absence of identifiable causes ("idiopathic") and they manifest as physiological responses in individuals responding to fear or stress [5-7].

Many known factors may increase the risk of developing GAD including stress, genetic factors, environmental factors, substance abuse, and physical or mental comorbidities (i.e. diabetes, depression, PTSD) [7]. Recent research also describes dysfunctional sympathetic nervous system signaling as a potential contributing factor in the development of GAD symptoms [8-10]. Cohort study data indicate augmented sympathetic nerve activity in response to chronic stress in anxiety sufferers compared to healthy individuals [8]. By using microneurography, brachial artery conductance, cardiorespiratory measures, and ambulatory 24-hr blood pressure to measure sympathetic nerve activity in both study groups, the results of this study indicate enhanced sympathetic nerve activity in individuals with chronic anxiety may be a modifiable risk or even causal factor in GAD [8].

Major Depressive Disorder (MDD) and Treatment Resistant Depression (TRD)

MDD is a psychiatric disease characterized by depressive symptoms, such as depressed mood, diminished interests, and impaired cognitive function. As at least 17 million US adults struggle with depression; understanding the pathophysiology of this disease is of utmost importance to the discovery of novel treatment options for this patient population [11]. In addition to a high prevalence rate, the presence of MDD is associated with an increased risk of developing other psychiatric disorders, such as GAD and PTSD [12-13]. Results from a prospective longitudinal cohort study over a 32-year duration exemplify the high comorbidity rates between GAD and MDD; of the 1037 individuals in the study cohort, 12% had comorbid GAD and MDD, but 72% of lifetime anxiety cases had a history of depression and 48% of lifetime depression cases had anxiety [12]. In addition, treatment-resistant depression (TRD), a subset of MDD, affects approximately 10%-30% of MDD patients, and individuals with TRD respond partially or not at all to traditional first-line antidepressant treatments, making this disease particularly difficult to treat [14].

As both the structural connections between neurons and between brain regions are formed and fine-tuned by the activity of neurotransmitters at neuronal synapses, alterations in neurotransmitter concentrations are widely accepted to play a role in the development and/or maintenance of depressive symptoms [12,15]. The focus of neurotransmitter-centric research has shifted from monoamines to glutamate [6-17], which has since been hypothesized to play a significant role in depression pathology [18-19]. Specifically, excessive glutamate signaling at N-methyl-D-aspartate receptors (NMDAR) may, at least in part, contribute to depression pathogenesis, and data have suggested that NMDAR dysfunction is associated with depression symptoms [18-19]. Another emerging model of depression pathophysiology extends beyond the role of neurotransmitters and focuses on functional connectivity and “circuit” disorders [20]. Additionally, brain-derived neurotrophic factor (BDNF), a “master-regulator” molecule with key roles in promoting neuroplasticity, expression is significantly reduced in patients with depression [21-24]. Further, animal models have revealed that depression-like behavior is associated with reduced BDNF expression in certain murine brain areas [21].

Given that traditional antidepressant treatments fail to provide relief for at least a subset of MDD patients (e.g. TRD populations) and the high comorbidity rates of MDD with other psychiatric disorders, designing therapeutic protocols for MDD has proven to be extremely complex due to the multifaceted nature of this condition (2-4,11). Despite this, in recent years MDD treatments have expanded significantly due to a greater understanding of its pathophysiology. With the approval of esketamine for TRD, and several different Transcranial Magnetic Stimulation (TMS) protocols (including the U.S. Food and Drug Administration breakthrough status for Stanford Neuromodulation Treatment), the array of potent and rapid-acting interventions with FDA approval is growing well beyond traditional “on the couch” psychiatric practice [25-26].

Post-traumatic Stress Disorder (PTSD)

PTSD is a debilitating psychiatric disorder that results from exposure to either real or perceived physical or mental injury/threat. PTSD is characterized by re-experience and avoidance symptoms such as intrusive thoughts, nightmares, flashbacks, dissociation, intense negative emotions, problems with sleep and concentration, irritability, increased reactivity, increased startle response, and hypervigilance [13]. As PTSD is one of the strongest correlates of suicidal ideation, lifetime suicide plans, and suicide attempts, PTSD has profound implications at the individual level and the global health level [27-28]. Individuals with PTSD are approximately 1.5 to 3 times more likely to experience co-occurring physical health conditions, such as diseases of bones and joints and neurological, respiratory, and cardiovascular illnesses [29]. Further, PTSD and GAD have at least an estimated 50% comorbidity rate in civilian populations and up to 91% comorbidity rate in veteran populations [30]. PTSD can significantly impair individual, social, and family functioning, and high rates of PTSD comorbidity with depression, GAD, substance abuse disorders, and physical health problems ultimately result in poor individual-level outcomes [13]. 

Despite many known risk factors for developing PTSD, the exact molecular mechanisms leading to PTSD pathogenesis are not completely understood. Research indicates that traumatic exposures lead to chronic and dysfunctional activation of the stress response pathways of the hypothalamic-pituitary-adrenal (HPA) axis and the sympathetic nervous system (SNS) [31]. HPA and SNS stress responses are orchestrated by neuroendocrine signaling between the autonomic nervous system (ANS) and target organs in the periphery [32]. The SNS and parasympathetic nervous system (PNS), the two branches of the ANS, are responsible for producing antagonistic physiological effects. Both the SNS and the PNS coordinate with the central nervous system through associated nerve ganglia, which act as a junction between the central nervous system and the sympathetic or parasympathetic nerve fibers innervating target organs in the periphery. In healthy, normally functioning individuals, SNS responses to stimuli are generally appropriate in magnitude and duration. In addition to internal or external physical stressors, mental or emotional stress has also been shown to stimulate the sympathetic nervous system and elicit similar physiological responses to physical stress [31-35]. Overstimulation of the SNS can lead to inappropriate physiological responses, and, unfortunately, physiological manifestations of sympathetic stimulation do not exclusively occur as a response to appropriate stimuli for individuals with PTSD [32,36]. Research has evidenced that patients with PTSD exhibit overactive sympathetic reactivity and activity both during mental stress and under resting conditions [27-28]. Overactivation of the SNS results in an abnormal release of glucocorticoids and catecholamines and elevated levels of glucocorticoids have been widely accepted to have downstream effects on negative feedback inhibition of the HPA axis [31]. As such, the abnormal release of glucocorticoids following exposure to traumatic events can lead to changes in neuroendocrine functioning (via increased glucocorticoid binding to glucocorticoid receptors) which maintains a hyperactive sympathetic state [31]. Multiple peer-reviewed publications have suggested that recurrent trauma-related symptoms experienced by PTSD patients (i.e. hyperarousal, heightened physiological responses to stressors, and increased startle responses) may arise from enhanced, prolonged, and/or inappropriate activation of the SNS stress response [21-22,30,37-38]. 

Furthermore, the current PTSD diagnosis does not fully capture the severe psychological harm that occurs when people experience repeated or prolonged traumatic exposure. Complex PTSD has recently been identified as a distinct condition by the International Disease Classification (ICD-11). Complex PTSD symptoms can be similar but more prolonged and extreme than those of PTSD and the ICD-11 diagnosis characterizes Complex PTSD according to symptoms of affect dysregulation, negative self-concept, and disturbed relationships [39]. Some mental health professionals are beginning to distinguish between the two conditions and echoing the urgent demands for more research on treating this condition, despite the lack of guidance from the Diagnostic and Statistical Manual of Mental Disorders, Fifth Edition (DSM-5) [40].

Metabolism and Psychiatric Disorders

While the presently available clinical data supports many hypotheses of the pathogenesis of mental disorders like PTSD, MDD, and GAD, it is important to note that emerging evidence highlights several shared common mechanistic bio-pathologies between metabolic dysfunction or abnormalities and such mental health disorders, including glucose hypo-metabolism, oxidative stress, inflammation, hormone abnormalities, and imbalances of neurotransmitter concentration or activity [41]. In recent years, research has revealed complex functions of molecules traditionally associated with metabolic disorders in the physiological systems that are involved in the development and/or maintenance of various psychiatric disorders. For example, in addition to its role in calcium and bone metabolism, Vitamin D is critical to numerous autocrine, paracrine, and endocrine processes which modulate the hypothalamic-pituitary-adrenal axis, a complex hormonal system that is implicated in several psychiatric disorders like PTSD, MDD, and GAD [42-43]. Further, as the vagus nerve, a major nerve of the parasympathetic nervous system, innervates the intestinal system and produces anxiolytic or anxiogenic signals in response to certain pathogenic bacteria in the gut microbiome, evidence suggests that the gut microbiome may influence brain function [44-45]. Numerous studies have demonstrated that patients with depression have distinct microbiota signatures when compared with healthy controls, highlighting a potential relationship between the gut microbiome and abnormal brain function [46]. Moreover, the effect of diet modification on symptom reduction of various psychiatric disorders has recently been studied to determine if dietary interventions with supposed psychopharmacologic properties could be used as potential treatments. Most notably, a 12-week randomized controlled trial investigating the effects of diet intervention on symptom severity in patients with depression reported significantly greater symptom improvement and significantly higher remission rates in the diet group relative to the control group [47]. 

Additionally, studies suggest that diet or lifestyle optimization and peptide therapy with anxiolytic peptides such as Selank, Semax, and PE 22-28 may serve as potential adjunct therapies for the treatment of several mental health disorders like PTSD, MDD, and GAD [47-51]. Although the evidence supporting a relationship between metabolic processes and the pathogenic mechanisms of various psychiatric disorders is biologically plausible, this field is relatively new and has thus been largely limited to evidence from animal studies and observational studies.

Current therapeutics (SGB, TMS therapy, and ketamine therapy)

Stellate Ganglion Block (SGB) and Dual Sympathetic Blocks (DSB)

Recent neuroscience research has revealed Stellate Ganglion Blocks (SGB) to be a promising new therapeutic avenue for individuals with PTSD and trauma-related anxiety. SGB is a minimally invasive procedure in which local anesthetic is injected into and around the cervical stellate ganglion nerve clusters under ultrasound guidance. Clinical studies have evidenced that SGB may provide significant and long-lasting symptom relief for patients with PTSD and trauma-related anxiety [30,52-59]. When used in conjunction with trauma-focused psychotherapy, SGB has been shown to have a 70%-80% success rate in treating PTSD symptoms [52-59]. A clinical case study involving 166 active duty service members with multiple combat deployments with PTSD who elected to receive an SGB demonstrated a <70% success rate of SGB, as measured by a reduction of at least 10 points in the PTSD Checklist for DSM-5 (PCL-5) symptom scores [58]. This study also reported the clinical benefits produced by SGB persisted beyond three to six months post-procedure in >70% of study participants [58]. Furthermore, a recent randomized clinical trial investigating SGB outcomes in patients with PTSD demonstrated that those who received SGB reported significantly improved PTSD assessment scores compared with patients in the placebo groups, as measured by a two times greater reduction in Clinician-Administered PTSD Scale for DSM-5 (CAPS-5) score [57]. This study also reported secondary outcome improvement in anxiety symptoms, as measured by a reduction in Generalized Anxiety Disorder 7-Item Scale (GAD-7) scores in PTSD patients [57].

These studies support the safety and efficacy of a standard right-sided, single-level (C6) SGB for the treatment of PTSD. In clinical practice, however, researchers noted some patients who did not respond to an SGB as anticipated, despite having similar clinical presentations to other patients that did respond well to the standard C6 SGB. To account for this discrepancy in clinical observation, a case study involving an analysis of 147 patients suggested treating two levels of the sympathetic chain to account for anatomic variations in the course of the cervical sympathetic chain and the location of the middle cervical ganglion [59]. This study treated 103 participants with a standard C6 SGB and 44 participants with a two-level SGB at C6 and C4 (herein referred to as Dual Sympathetic Blocks or DSB). The mean baseline PCL scores for each group were compared to one another and both groups experienced clinically significant improvement in PTSD symptoms (i.e. a PCL score reduction of at least 10 points). The (single-level) SGB group responded with a mean improvement in the PCL-5 score of 25.2 points, while the DSB group responded with a greater improvement of 31.78 points [59]. Based on this initial clinical report for the two-level cervical sympathetic chain block (DSB) treatment modality, a right-sided two-level cervical sympathetic chain block (DSB) administered at C6 and C4 levels appears to be safe and more effective than a standard SGB in the treatment of PTSD symptoms.

Normal anatomic variation along the course of the cervical sympathetic chain and in the location of the middle cervical ganglion reasonably supports the DSB approach. Furthermore, research suggests a left-sided SGB has different effects than a right-sided SGB. A large analysis [58] of blood pressure (BP) and heart rate (HR) changes after right and left SGBs indicated higher sympathetic dominance on the right while higher parasympathetic dominance on the left side. Additionally, a retrospective study [56] of 205 PTSD patients found 20 who did not respond to a right SGB and subsequently treated 10 of those patients with a left-sided SGB. This resulted in clinically significant improvement where 90% responded favorably to the left-sided SGB and the mean PCL-5 improvement was 28.5 points [56]. For these reasons, the preferred modality here is the DSB approach performed on both the right and left sides, as this approach has demonstrated consistent and superior clinical efficacy when compared to a single-level, single-sided block.

Ketamine Therapy

In recent years, overwhelming evidence has substantiated the potential benefits of ketamine in treating psychiatric disorders, particularly depression and chronic pain [50-51]. Ketamine can provide rapid and significant improvements in depressive symptoms, even for patients with treatment-resistant depression [50-51]. Ketamine is a drug classified as a dissociative anesthetic hallucinogen because it exerts its biochemical actions on the glutamatergic system. Tight regulation of extracellular glutamate levels is of utmost physiological importance because glutamate is found in extremely high concentrations and the excitatory effects of glutamate are very potent [52]. Glutamate acts on two classes of receptors, ionotropic glutamate receptors, including N-methyl-D-aspartate (NMDA), α-amino-3-hydroxy-5-methyl-4-isoxazole propionic acid (AMPA), and kainate (KA), as well as metabotropic glutamate receptors [52]. In healthy brains, glutamate signaling through NMDARs contributes to neuroplasticity [18]. NMDAR dysfunction is associated with depression [18-19]. Data has also suggested that excessive glutamate signaling at NMDA receptors may, at least in part, contribute to depression pathogenesis [18,34]. Ketamine disrupts the glutamatergic system and blocks the activity of NMDA receptors on gamma-aminobutyric acid (GABA)ergic interneurons, leading to downstream increases in extracellular glutamate levels [52].

Reports from numerous clinical trials have demonstrated that a single intravenous ketamine infusion can produce a significantly rapid and sustained antidepressant response [53-54]. The mechanism by which ketamine improves depressive symptoms is not completely understood, but, in addition to affecting NMDAR signaling, ketamine may affect AMPA receptors (AMPARs), a different class of ionotropic glutamate receptors. Glutamine action at AMPAR is responsible for modifying synaptic strength and supporting cellular remodeling in response to learning [55-56]. AMPA receptors and NMDA receptors are antagonists; activation of AMPAR results in the inhibition of further glutamate release and reduces glutamate activity [21-22]. Ketamine modulates glutamate activity in the brain in two ways: by blocking NMDA receptors and by activating AMPA receptors [21]. Ketamine has been shown to block NMDA ion channels [18,21]. Ketamine-mediated blockade of NMDA ion channels causes glutamate release from NMDAR into the extracellular space, effectively “freeing” glutamate to act at AMPA receptor sites [18,21]. The subsequent binding of glutamate to AMPAR may be partly responsible for the antidepressant effects of ketamine by inducing inhibition of glutamate recycling and release [21].

Additionally, ketamine may also elicit antidepressant effects by modulating the production of neurotrophic factors such as brain-derived neurotrophic factor (BDNF) [12-24,57-58]. BDNF expression has been shown to be significantly reduced in patients with depression, and several meta-analyses have revealed that patients with depression exhibit significantly reduced blood BDNF levels [21-23]. Animal models of stress have demonstrated that depression-like behavior is associated with reduced BDNF expression in certain murine brain areas [21]. It has been hypothesized that ketamine significantly increases BDNF expression through AMPAR-mediated activation of the mTOR pathway [24]. Evidence now supports that BDNF is required for and may partly mediate the significant and rapid antidepressant effects of ketamine [22-24,57-58].

As a powerful regulator of glutamate, ketamine has many clinical applications in expanding treatment options for mental health disorders like depression. In particular, the incredibly rapid onset of antidepressant effects that ketamine infusions produce is of utmost clinical importance to patients with depression suffering from suicidal ideation. A recent meta-analysis concluded that single-dose intravenous ketamine infusions remarkably reduce patients' suicidal thoughts as soon as two hours after infusion [58]. These results highlight ketamine as a fast-acting and successful therapeutic avenue for individuals struggling with severe depression. In addition, approximately 40% of patients with treatment-resistant depression (TRD) present with cognitive deficits [59]. The results from another recent systematic review suggest that TRD patients treated with ketamine infusions showed improved complex and simple working memory, improved processing speed, and improved verbal learning memory [59]. These results provide further evidence for the safety and efficacy of therapeutic ketamine in depression treatment protocols.

Transcranial Magnetic Stimulation (TMS) Therapy

Conventional treatments for MDD include psychological therapy methods and pharmacological antidepressant medications such as selective serotonin reuptake inhibitors, serotonin and noradrenaline reuptake inhibitors, tricyclic antidepressants, and electroconvulsive therapy [60]. Recent discoveries in depression treatment research have revealed TMS therapy as a novel therapeutic option with significantly fewer potential adverse effects. TMS therapy is a non-invasive treatment option with an excellent safety and efficacy profile that has been designed to provide significant and long-lasting symptom relief from numerous psychiatric disorders like depression, migraines, and obsessive-compulsive disorder [61-62].TMS is a noninvasive brain stimulation therapy that involves the application of targeted magnetic pulses to the superficial layers of the cerebral cortex. This magnetic field can locally induce small electrical currents that stimulate nerve cells in mood-controlling areas of the brain [11,61-64]. These small electrical currents are both powerful and precise enough to elicit an action potential in neurons in the frontal lobe, hippocampus, temporal lobe, thalamus, striatum, and amygdala, effectively resulting in increased release of neurotransmitters into the synapse [61-64]. In patients with depression, alterations in functional connectivity are prominent, and the use of functional connectivity for TMS targeting is part of the workflow for neuronavigated TMS, as described in the pivotal trial by Cole et al. for Stanford Neuromodulation Treatment [25]. TMS therapy addresses the neurotransmitter imbalance associated with depression by stimulating targeted neurotransmitter release in mood-controlling regions of the brain [61-62]. TMS therapy may restore neuronal circuit activity in patients with depression and has been shown to provide statistically and clinically significant improvement of depressive symptoms [61-67]. Symptom improvement in mood, reduced days of experiencing depressive symptoms and increased engagement in socializing have been reported as early as two weeks following the completion of the TMS therapy protocol [68]. Approximately 83% of patients treated with TMS therapy showed significant improvements in depressive symptoms, and 62% of patients reported symptom relief lasting through 12 months [66-67,69].

The IMH approach

Results from myriad clinical trials support the safety and efficacy of each of the three therapeutic avenues included in our proposed Interventional Mental Health subspecialty for the management of various psychiatric disorders. Extensive data from clinical studies evaluating Stellate Ganglion Blocks (SGB) suggest these procedures may provide clinically meaningful and long-lasting symptom relief for patients with PTSD and trauma-related anxiety. Intravenous ketamine therapy has been repeatedly shown to provide rapid, significant, and long-lasting improvements in depressive symptoms, even for patients with TRD. Further, TMS therapy has also been shown to provide a marked improvement in depressive symptoms in patients with MDD. 

Given that SGB, intravenous ketamine therapy and TMS therapy utilize distinct molecular and physiological pathways to achieve clinically meaningful symptom relief of PTSD, anxiety, and depression and the notably high comorbidity rates between these disorders, employing a combination of treatment modalities to treat multiple comorbidities simultaneously through an IMH approach may improve therapeutic effectiveness and efficiency. We hypothesize that creating unique treatment algorithms which combine these therapeutic approaches in a consolidated time frame will produce synergistic outcomes in at least a subset of patients with comorbid PTSD, depression, and/or anxiety. Implementing IMH-centered treatment protocols involving a combination of DSB and/or intravenous ketamine therapy and/or TMS therapy will provide valuable insight into understanding what combination of therapeutic modalities is the most efficacious for certain conditions. 

Clinical trial data suggests two SGB treatments performed a maximum of two weeks apart are most effective in reducing PCL-5 scores and significantly improving PTSD symptoms [46]. The antidepressant effects of a single dose of intravenous ketamine persist for a maximum of seven days, so numerous studies have explored the safety and efficacy of repeated intravenous ketamine infusions over several study periods (e.g. three to six times a week for one to six weeks). Among these, one study evaluated the antidepressant effects of a series of six intravenous ketamine (0.5mg/kg) infusions in patients with TDR three times weekly over a 12-day study period and reported an overall response rate of 70.8% [70]. The authors also noted a significant reduction in Montgomery-Åsberg Depression Rating Scale (MADRS) scores two hours after the first ketamine infusion, which was also largely sustained throughout the study [70]. As such, presently available clinical data indicates a series of six intravenous ketamine sessions administered over two weeks is required for prolonged antidepressant effects. Likewise, five daily treatment sessions over three to six weeks (a total of 20 to 30 sessions) of TMS therapy are required for effective and prolonged clinical benefits for patients with MDD [71]. As evidence strongly supports the safety and efficacy of SGB, ketamine therapy, and TMS therapy as independent treatment modalities, one would reasonably anticipate that sequential administration of these therapies could provide synergistic and potentiated clinical benefits over a condensed time frame.

The IMH approach we propose begins by performing a unilateral DSB directly followed by intravenous ketamine therapy - all in the span of a single appointment. We will administer the ketamine infusion 40 minutes after the SGB is performed to minimize any interaction between ketamine and propofol, the anesthetic we use for sedation. We hypothesize that merging the time frame in which patients receive these treatments will potentiate the therapeutic benefits of each individual therapy. In our clinical practice, we have observed that some patients with moderate to severe anxiety can occasionally experience unpleasant intravenous ketamine therapy sessions due to the dissociative effects of ketamine. As such, we believe performing DSB before intravenous ketamine therapy may reduce excessive sympathetic signaling during ketamine therapy sessions because DSBs have been shown to alleviate symptoms of sympathetic reactivity and anxiety-related PTSD symptoms, which are frequently concomitant with depression. Additionally, research suggests that DSBs improve cerebral perfusion through vasodilation mechanisms, which may aid in the efficacy of ketamine therapy and TMS therapy (80). If performed consecutively over the duration of a one-week treatment intensive, we hypothesize that two DSBs followed by six intravenous ketamine sessions and the completion of the Stanford Accelerated Intelligent Neuromodulation Therapy (SAINT) accelerated TMS therapy protocol may lead to better patient experiences and improved overall outcomes.

With regard to the suitability of the IMH approach for different age groups, in our clinical practice, we have successfully treated pediatric psychiatric patients as young as eight years old with SGB procedures under the multidisciplinary care team of a child and adolescent psychiatrist, anesthesiologist, and their treating therapist. In many of these younger patients with significant psychiatric issues, polypharmacy approaches have often been trialed with inadequate efficacy. Some of the modalities presented by the IMH approach (i.e. SGB and TMS therapy) may offer a novel, non-pharmacologic treatment approach for a subset of these patients. While there are several published case reports that describe safe and efficacious results of SGB in pediatric patients as young as 18 months old, supporting evidence from clinical trials is not yet available [72-74]. Further, the safety and efficacy of ketamine therapy in adolescent populations between the ages of 12 and 18 with depression have been demonstrated across numerous clinical studies, indicating that ketamine therapy may be appropriate not only for adults with depression but also for some adolescents with depression [75-76]. Additionally, TMS therapy has been demonstrated to be safe and effective in pediatric and adolescent psychiatric populations from 12-18 years old across various clinical studies [77-78]. Potential contraindications and side effects of SGB procedures (i.e. risk of infection, bleeding, nerve damage to the vagus nerve, recurrent nerve or brachial plexus roots, intolerability secondary to pain), intravenous ketamine therapy (i.e. psychomimetic effects that lead to long-term cognitive deficits, the potential for abuse and dependence) and TMS therapy (i.e. seizure) can be minimized in the proposed IMH approach because the multidisciplinary nature of this novel medical discipline calls for the collaboration of highly trained medical professionals in their respective fields who can contribute their specific skill sets appropriately.

Limitations

While the IMH approach may be successful in creating a targeted mental health treatment modality, it may be cost-ineffective, primarily due to the lack of readily available insurance coverage. Moreover, the need for a definitive certainty regarding how to precisely structure a combined intervention treatment protocol still remains. Additionally, the scarcity of medical professionals equipped with the requisite training and suitable practices to support this approach contributes to its limitations. As such, these factors collectively underscore the need for specialized expertise to inform practice standards for treatment protocols as well as the cost-effectiveness of the IMH approach relative to other treatment modalities. Additionally, though we hypothesize that the combination of treatments in the IMH approach is potentially synergistic in mechanisms of action to yield additive clinical efficacy, randomized controlled trials are needed to conclusively determine whether that additive effect is actual.

Strengths and implications

This paper proposes the utility of combing interventional mental health treatments to improve treatment efficacy for select psychiatric conditions, particularly in treatment-resistant patient populations, as well as adding clinically indicated combinations to practice standard treatment protocols. The implications of this research are significant, expanding the range of interventions for treatment-resistant populations, and also offering an opportunity for swifter symptom improvement to subsequently improve engagement with and efficacy of conventional treatment modalities.

## Conclusions

The implications of this research could be significant, as it offers a promising alternative for individuals whose conditions have been challenging to address using conventional treatments alone. If proven effective, this IMH model could expand treatment options, offering hope to patients who previously had limited therapeutic avenues. Furthermore, our emphasis on utilizing a consolidated timeframe for administering different therapeutic modalities could lead to more efficient treatment, reducing the overall duration of interventions and potentially minimizing the burden on both patients and healthcare resources.

Overall, the IMH focus on combining diverse therapeutic approaches to address complex mental health conditions showcases a forward-thinking and novel approach to mental healthcare. By exploring new avenues and testing the synergistic potential of different interventions, this research contributes to the ongoing efforts to improve mental health treatment and, ultimately, the well-being of affected individuals.
